# Influence of Health Insurance Coverage on the Survival Rate for Primary Total Knee Arthroplasty: Minimum 5-Year Follow-Up Analysis

**DOI:** 10.3390/healthcare12161601

**Published:** 2024-08-12

**Authors:** Jae-Sung Seo, Jung-Kwon Bae, Seong-Kee Shin, Hyung-Gon Ryu, Kyu Jin Kim, Seung Yeon Cho

**Affiliations:** Department of Orthopedic Surgery, Seoul Medical Center, Seoul 02053, Republic of Korea

**Keywords:** arthroplasty, replacement, knee, survival rate, health insurance coverage

## Abstract

This study investigated whether differences in survival rates and clinical outcomes exist in patients undergoing TKA by insurance type: National Health Insurance (NHI) vs. Medical Aid Program (MAP). This study conducted a retrospective analysis of 762 TKAs (NHI, *n* = 505; MAP, *n* = 257) with a mean follow-up of 8.4 ± 1.8 years. Patient-reported outcomes (PROMs) were evaluated using the American Knee Society’s (AKS) score at the final follow-up. The survival rate of each group was analyzed using Kaplan–Meier survival analysis. Any postoperative complications and readmissions within 90 days of discharge were recorded and compared between the groups. There were no between-group differences in pre- to postoperative improvement in AKS scores. The estimated 10-year survival rates were 98.5% in the NHI group and 96.9% in the MAP group, respectively, with no significant differences (*p* = 0.48). However, the length of hospital stay (LOS) was significantly longer in the MAP group than in the NHI group (13.4 days vs. 13.1 days, *p* = 0.03), and the transfer rate to other departments was significantly higher in the MAP group than in the NHI group (3.9% vs. 1.4%, *p* = 0.04). Readmission rates for orthopedic complications for 90 days were 3.0% in the NHI group and 3.5% in the MAP group, respectively (*p* = 0.67). Patients’ insurance type showed similar survival rates and clinical outcomes to those of primary TKA at a mean follow-up of 8.4 years, but the LOS and rate of transfer to other departments during hospitalization were influenced by insurance type.

## 1. Introduction

Total knee arthroplasty (TKA) is a well-established treatment to reduce pain and enhance function among individuals suffering from end-stage arthritis [1]. Although TKA utilization has increased exponentially worldwide over the last two decades, socioeconomic status (SES) has been an important factor leading to varied effects on the treatment timing, type of treatment, and patient health status, thereby contributing to persistent disparities in utilization [2,3,4,5]. Health insurance coverage, which is determined by employment status and financial resources, is an effective indicator of SES [6] and can affect the outcomes after TKA. Few studies have examined the correlation between patient insurance type and clinical outcomes after TKA [7,8]. Studies indicate that Medicaid patients undergoing TKA often experience extended lengths of stay, higher likelihoods of discharge to rehabilitation facilities, and increased readmissions within 90 days [9,10]. Additionally, the Medicaid population encounters higher risks of inpatient mortality and postoperative complications, leading to poorer postoperative outcomes [8,11,12]. However, these studies included patients who underwent TKA over a short follow-up period at multiple centers [7,8,9,10,11,12]. Moreover, there is a paucity of literature on the impact of health insurance coverage on implant survival. As such, we investigate whether differences in implant survivorship and clinical outcomes exist in patients who received TKA performed at a single institution when stratified by insurance type. We answer three questions: (1) Does health insurance type influence patient-reported outcomes (PROMs)? (2) Does health insurance type influence implant survival? And (3) Do orthopedic/medical complications differ depending on insurance type?

## 2. Materials and Methods

### 2.1. Health Insurance Coverage

The National Health Insurance (NHI) and Medical Aid Program (MAP) are the two major healthcare systems in South Korea [13]. The NHI requires individuals to pay insurance premiums for healthcare coverage. The MAP provides low-cost or free healthcare services to economically disadvantaged individuals. Both systems ensure access to healthcare but differ in terms of funding and eligibility criteria [14,15]. The NHI ensures a wide range of healthcare services, including general medical care, preventive vaccinations, dental treatments, traditional medicine, hospitalization, and surgery. Most healthcare services are covered by insurance, and patients bear some of the costs themselves. MAP provides basic medical services and various forms of social support, such as specific disease treatments, medication subsidies, and assistive devices for disabilities.

### 2.2. Study Design and Patients

**Figure 1:**
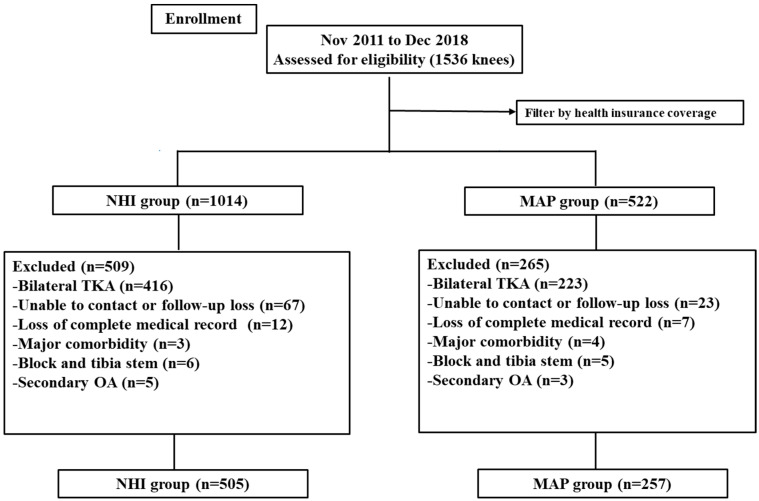
Flow diagram showing the number of knees that met the study criteria. NHI, National Health Insurance; MAP, Medical Aid Program.

We conducted a retrospective collection of clinical data from consecutive patients who received TKA at our institute from November 2011 to December 2018. Inclusion criteria were as follows: patients (1) with symptomatic progressed primary osteoarthritis (Kellgren–Lawrence grade ≥ III) [16] and (2) who received unilateral TKA using a cemented posterior-stabilized type. The exclusion criteria were patients with loss of complete medical records and those who were followed up for less than 5 years. Patients with significant comorbidities, including severe pulmonary disorders, coagulation abnormalities, or a history of venous thromboembolism, were excluded. To minimize bias, patients with a second knee in staged bilateral TKA, secondary osteoarthritis (OA), or stem/block fixation for primary TKA were excluded from this study. Finally, 762 knees were assigned to either the NHI group (*n* = 505) or the MAP group (*n* = 257) (Figure 1). The study protocol received approval from our institutional review board (SMC 2022-01-009).

### 2.3. Surgical Methods and Postoperative Management 

All surgical procedures were executed by a single surgeon (SJS) within the confines of one institution, and each patient received care in adherence to the same protocol. A pneumatic tourniquet was utilized during the operation and deflated after the surgical procedure ended. The surgical procedure was performed using the modified gap balance technique [17]. A medial parapatellar arthrotomy was used through the use of a midline incision. Following the meticulous removal of osteophytes from both the femur and tibia, an intramedullary alignment rod was employed to establish the angle for the distal femur cut, with the aim of a valgus alignment between 5° and 6°. A standard extramedullary jig was used on the proximal tibia to ensure a cut perpendicular to the tibial axis. Rotation of the femoral component was determined based on an assessment of the balanced flexion gap. Femoral size was measured using an anterior reference system to ensure procedural consistency and accuracy. A suction drain was subsequently inserted into the knee joint and unclamped upon placement. Following surgery, all patients were fitted with compressive elastic stockings for four weeks postoperatively to prevent deep vein thrombosis (DVT). Prophylactic antithrombotic treatment was not used due to potential drug-induced bleeding complications. Postoperative rehabilitation commenced on day 1, including bedside continuous passive mobilization, range of motion (ROM) exercise, calf-pump exercises, and straight leg raising. The suction drain was removed 72 h postoperatively, and patients were encouraged to ambulate based on their tolerance level.

### 2.4. Clinical Evaluations and Survival Rate

Clinical assessment utilized the American Knee Society (AKS) score [18]. Subsequent follow-up assessments were scheduled at the outpatient department at intervals of 6 weeks, 3 months, 6 months, and 1 year post-surgery. Clinical and radiographic evaluations were conducted at each visit. Postoperative complications and readmissions within 90 days after discharge were recorded separately for medical and orthopedic reasons and then compared between the groups. Moreover, the number of patients transferred to other departments during hospitalization for medical reasons was recorded. Occurrences of wound dehiscence, stiffness, and hemarthrosis were documented. Hemarthrosis was assessed by evaluating the patellar circumference. Acute postoperative periprosthetic joint infection (PJI) diagnosis adhered to the criteria based on the International consensus meeting in 2018 [19]. Stiffness was characterized by a flexion limitation of around 95°, hindering the patient’s ability to engage in various activities. Symptoms indicating potential pulmonary embolism, such as shortness of breath, blood-streaked sputum, or chest pain, were assessed during computed tomography (CT) for pulmonary thromboembolism (PTE) CT. Implant survival rates over a 10-year period were also evaluated. The primary endpoint was revision TKA for implant-related complications or infections. Failure attributed to infection was defined as septic failure, while failure due to other causes was defined as aseptic failure.

### 2.5. Statistical Analysis

The normality of variables was assessed using the Kolmogorov–Smirnov test, while comparison of continuous data utilized an independent samples *t*-test. Categorical data were compared using either Chi-squared or Fisher’s exact tests. Kaplan–Meier survival analysis was employed to estimate the 10-year survival rate for each group, with survival curves compared using a log-rank test. Statistical significance was defined as a two-sided *p*-value below 0.05. All statistical analyses were conducted using SPSS software version 23 (IBM Corp, Armonk, NY, USA), and continuous data were presented as mean ± standard deviation.

## 3. Results

A total of 762 patients (505 with NHI and 257 with MAP) with a mean age of 72.8 ± 6.6 years and follow-up of 8.4 ± 1.8 years were included in this study. Patient demographic characteristics are summarized in Table 1. There were no significant demographic differences between the groups, except for the length of hospital stay (LOS). The LOS was significantly longer in the MAP group than in the NHI group (13.4 days vs. 13.1 days, *p* = 0.03). The prosthetic components are listed in Table 2.

When we compared the preoperative AKS scores between the two groups, the MAP group (knee, 25.1 ± 12.1; function, 23.5 ± 11.9) had significantly lower scores than the NHI group (knee, 31.9 ± 12.5; function, 29.8 ± 11.6, *p* = 0.001). However, the degree of improvement in the postoperative AKS knee and function scores compared to the preoperative values was similar between the groups. The degree of improvement in the range of motion (ROM) was also no different between the groups (Table 3).

**Figure 2:**
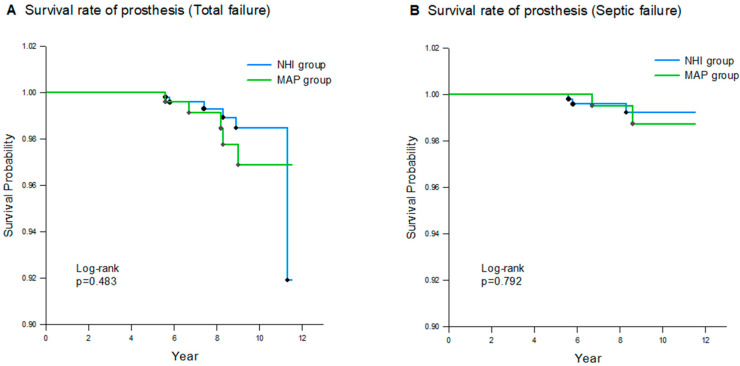
Kaplan–Meier analysis of cumulative survival for all-cause failure of NHI group and MAP group: (**A**) failure due to all complications; (**B**) septic failure (due to infection).

Survival rates at the 10-year follow-up were 98.5% and 96.9% for the NHI and MAP groups, respectively. The survival rates for septic failure were also calculated. In the septic failure group, the 10-year survival rates were 99.2% and 98.7% in the NHI and MAP groups, respectively. A log-rank test was performed to analyze the differences in survival curves between the groups, and no statistically significant differences were found (*p* = 0.48 for total failure; *p* = 0.79 for septic failure) (Figure 2).

There were no significant differences in orthopedic complications and 90-day readmission between the groups (Table 4). The 90-day readmission for orthopedic complications appeared in 24 knees: 15 in the NHI group and 9 in the MAP group (*p* = 0.67). Similarly, there were no significant differences in medical complications and 90-day readmission between the two groups (Table 5). The 90-day readmission for medical complications appeared in fourteen knees: seven in the NHI group and seven in the MAP group (*p* = 0.25). However, the rate of transfer to other departments during hospitalization was significantly higher in the MAP group than in the NHI group (3.9% vs. 1.4%, *p* = 0.04).

## 4. Discussion

The main finding was that health insurance coverage showed similar survival rates and clinical outcomes of primary TKA at a mean follow-up of 8.4 years, while LOS and the rate of transfer to other departments during hospitalization were influenced by health insurance coverage. Considering the varied impacts of SES on patient status, it is noteworthy that the rates of prosthesis survival, improvement of patient-reported outcome measures (PROMs), and occurrences of orthopedic complications are similar among patients with different insurance types. Although further research is necessary to establish a causal relationship, these findings deserve careful consideration by arthroplasty surgeons counseling patients with MAP.

Based on the theoretical advantages of TKA, we hypothesized that comparable results, similar to those observed in individuals with NHI, could be achieved in individuals with MAP. However, Martin et al. [20] conducted a retrospective review of 293 consecutive patients who underwent total joint arthroplasty, stratifying them based on their insurance type. Medicaid coverage, similar to MAP, was a significant predictor of lower preoperative and postoperative PROMs [21]. Hinman and Bozic retrospectively reviewed 224 patients undergoing total hip arthroplasty and reported lower preoperative and postoperative Harris hip scores in Medicaid-covered patients than in non-Medicaid-covered patients [22]. These findings are thus consistent with those of previous studies. Our findings showed that postoperative AKS scores were significantly lower in the MAP group compared to the NHI group at the latest follow-up. Additionally, preoperative AKS scores were also significantly lower in the MAP group, which seems reasonable considering that MAP patients are likely less educated, engage in physically demanding situations, live alone, smoke tobacco, have poor hygiene, and have a lower baseline, as measured by PROM. Therefore, the difference in postoperative AKS scores between the two groups could be attributed to the initial difference in the preoperative AKS scores between the groups. Furthermore, there were no significant differences observed between the two groups regarding the postoperative improvement in the AKS scores compared to their preoperative values. Thus, when using PROMs to evaluate the value of care, preoperative to postoperative changes serve as more reliable indicators of surgical success than absolute values, especially in patients with MAP [23].

Although several studies have examined the relationship between socioeconomic factors and outcomes for TKA [2,3,4], few studies evaluated the influence of socioeconomic factors such as insurance payer type on TKA survival. Many studies have investigated the survivorship of primary TKA at 10 years and found survival rates of 92–98% [24,25,26]. Our study showed that the survival rates at the 10-year follow-up were 98.5% and 96.9% for the NHI and MAP groups, respectively. The higher survival rates in this study can be attributed to the fact that the literature on survivorship of primary TKA mainly comprises studies conducted from the late 2000s to the early 2010s [24,25,26], which reported comparatively lower survival rates than current studies. Moreover, surgical techniques and component designs have evolved in recent years, with a focus on improving function and outcomes after TKA [27,28]. We also confirmed that there was no statistically significant difference in the 10-year survival rates between the two groups. We believe that these results are due to the characteristics of public healthcare hospitals, where MAP patients tend to have longer hospital stays and there is an effective multidisciplinary approach within the healthcare system for the treatment of MAP patients [29].

There was no significant difference in orthopedic complications between the two groups during the midterm follow-up. Similarly, there were no differences in medical complications between the groups during hospitalization. However, the transfer rate to other departments during hospitalization was significantly higher in the MAP group. These factors contributed to the extended LOS for the MAP group. Moreover, in the case of the NHI group, additional private insurance is often purchased, and the hospitalization period may vary depending on the support of the terms and conditions of the purchased private insurance. However, for the MAP group, it may be easy to extend the hospitalization period within the support amount because the MAP group receives a fixed amount and there are many systems or assistance for receiving additional support. An extended LOS may enable a multidisciplinary team to plan a patient-tailored rehabilitation path and better allocate resources to maximize recovery [29,30]. Skura et al. [31] reported that the LOS following total joint arthroplasty was influenced by insurance type. Similarly, in this study, the LOS was affected by insurance type. The authors believe that the individuals in the MAP group, who are socially disadvantaged, undergo basic examinations before surgery but are more likely to have underlying conditions than those in the NHI group [14,15]. These patients are at higher risk of perioperative complications, leading to a higher rate of transfer to other departments [32].

Our study is not without limitations. First, this was a retrospective study with a relatively small number of patients in the MAP group. Considering that the qualifications for MAP are strict, it would be difficult to proceed with such a large number of cases prospectively. Since this study is a study on mid-term follow-up, further research may be necessary to study long-term follow-up in the future. Further, our study was conducted by a single surgeon, and we used a posterior-stabilized knee design for all patients, who adhered to the same rehabilitation protocols at a single institute. Second, several implants were used, but there was no statistical significance between implant use. Moreover, although we used different implants, all implants are widely used and proven without differences in the market. Therefore, bias in the results was likely minimal.

## 5. Conclusions

We should cautiously consider the fact that the patients’ insurance type showed similar survival rates and clinical outcomes to those of primary TKA at a mean follow-up of 8.4 years, but the LOS and the rate of transfer to other departments during hospitalization were influenced by the patients’ insurance type.

## Figures and Tables

**Table 1:** Patients’ demographic characteristics.

Variables	NHI Group (*n* = 505)	MAP Group (*n* = 257)	*p*-Value
Sex (male/female)	93/412	54/203	0.391
Mean age (years)	73.0 ± 6.7	72.5 ± 6.4	0.386
Mean BMI (kg/m^2^)	27.1 ± 5.3	27.4 ± 5.3	0.375
Mean ASA score			0.214
1	40 (7.9)	17 (6.6)	
2	377 (74.7)	182 (70.8)	
3	88 (17.4)	58 (22.6)	
Comorbidity			0.527
Hypertension	377 (74.7)	207 (80.5)	
Diabetes mellitus	243 (48.1)	143 (55.6)	
Angina	74 (14.7)	51 (19.8)	
Stroke/CVA	47 (9.3)	28 (10.9)	
CKD	24 (4.8)	21 (8.2)	
Preoperative HKA angle (°)	−8.2 ± 6.4	−8.9 ± 6.6	0.661
Preoperative K-L grade (3/4)	78/427	42/215	0.748
Length of hospital stay (days)	13.1 ± 0.9	13.4 ± 1.6	0.031
Follow-up period (years)	8.3 ± 1.8	8.5 ± 1.8	0.176

Values are presented as mean ± standard deviation or numbers (%). NHI, National Health Insurance; MAP, Medical Aid Program; BMI, body mass index; ASA, American Society of Anesthesiologists; CVA, cerebrovascular accident; CKD, chronic kidney disease; HKA angle, hip–knee–ankle angle (negative values indicating varus alignment); K-L grade, Kellgren–Lawrence grade.

**Table 2:** The types of prosthetic components used.

Implant	NHI Group	MAP Group	*p*-Value ^a^
	(*n* = 505 Knees)	(*n* = 257 Knees)	
			0.002
Vanguard, Biomet	105 (20.8)	76 (29.6)	
Scorpio NRG, Stryker	112 (22.2)	67 (26.1)	
Genesis, Smith-nephew	142 (28.1)	57 (22.2)	
Optetrak, Exactech	84 (16.6)	22 (8.7)	
Lospa, Corentec	62 (12.3)	35 (13.6)	

Values are presented as numbers (%). NHI, National Health Insurance; MAP, Medical Aid Program. ^a^ Chi square test.

**Table 3:** Clinical outcomes between the groups.

	NHI Group (*n* = 505)	MAP Group (*n* = 257)	*p*-Value ^a^
Preoperative			
AKS knee score	31.9 ± 12.5	25.1 ± 12.1	<0.001
AKS function score	29.8 ± 11.6	23.5 ± 11.9	<0.001
FC (°)	8.6 ± 8.3	8.9 ± 8.7	0.623
FF (°)	124.8 ± 10.5	125.1 ± 9.3	0.620
Postoperative			
AKS knee score	92.3 ± 4.8	85.0 ± 4.8	<0.001
AKS function score	92.4 ± 4.7	85.5 ± 4.6	<0.001
FC (°)	1.2 ± 2.5	1.4 ± 2.5	0.649
FF (°)	129.0 ± 2.7	128.9 ± 3.1	0.176
Improvement from preoperative			
AKS knee score	60.4 ± 13.4	60.0 ± 12.8	0.659
AKS function score	62.5 ± 12.9	62.0 ± 12.3	0.575
FC (°)	7.4 ± 7.9	7.5 ± 8.6	0.879
FF (°)	4.2 ± 9.9	3.8 ± 8.8	0.509

Values are presented as mean ± standard deviation. NHI, National Health Insurance; MAP, Medical Aid Program; AKS, American Knee Society knee score; FC, flexion contracture; FF, further flexion. ^a^ Student’s *t*-test was used to compare continuous variable outcomes between the groups.

**Table 4:** Comparison of orthopedic complication between the groups.

Variables	NHI Group (*n* = 505 Knees)	MAP Group (*n* = 257 Knees)
Stiffness	5 (1)	2 (0.8)
Hemarthrosis	3 (0.6)	1 (0.4)
Proximal DVT	4 (0.8)	2 (0.8)
Patella tendon rupture	2 (0.4)	1 (0.4)
Cellulitis (away from surgical site)	7 (1.4)	4 (1.6)
Wound dehiscence	10 (2.0)	5 (1.9)
Superficial SSI	7 (1.4)	4 (1.6)
PJI	3 (0.6)	2 (0.8)
Aseptic loosening	2 (0.4)	2 (0.8)
Dislocation/instability	1 (0.2)	1 (0.4)
Periprosthetic fracture	5 (1)	3 (1.2)
90-day readmission	15 (3.0)	9 (3.5)

Values are presented as numbers (%). NHI, National Health Insurance; MAP, Medical Aid Program; DVT, deep vein thrombosis; SSI, surgical site infection; PJI, periprosthetic joint infection.

**Table 5:** Comparison of medical complications between the groups.

Variables	NHI Group (*n* = 505 Knees)	MAP Group (*n* = 257 Knees)	*p*-Value ^a^
Pneumonia	3 (0.6)	5 (1.9)	0.127
Pulmonary embolism	1 (0.2)	1 (0.4)	1
Stroke/CVA	2 (0.4)	3 (1.2)	0.342
Myocardial infarction	2 (0.4)	3 (1.2)	0.342
Sepsis	1 (0.2)	1 (0.4)	1
Death	1 (0.2)	2 (0.8)	0.264
Transferred to other departments	7 (1.4)	10 (3.9)	0.037
90-day readmission	7 (1.4)	7 (2.7)	0.253

Values are presented as numbers (%). NHI, National Health Insurance; MAP, Medical Aid Program; CVA, cerebrovascular accident. ^a^ Fisher’s exact test.

## Data Availability

The raw data supporting the conclusions of this article will be made available by the authors on request.
